# Subcuticular sutures versus staples for skin closure in patients undergoing abdominal surgery: A meta-analysis of randomized controlled trials

**DOI:** 10.1371/journal.pone.0251022

**Published:** 2021-05-04

**Authors:** Juntao Feng, Xiaoli Jiang, Zhifu Zhi

**Affiliations:** Department of Gynecology and Obstetrics, The First Affiliated Hospital of Guangxi Medical University, Nanning, People’s Republic of China; University of Insubria, ITALY

## Abstract

**Background:**

Surgical site infections (SSIs) are common postoperative complications. Whether the use of staples or sutures makes a difference in abdominal surgery’s infection rate remains elusive.

**Methods:**

A systematic review was performed to identify randomized clinical trials comparing staples and sutures after abdominal surgeries. Eligibility criteria involved the SSI occurrence as the primary outcome and the incidence of wound dehiscence, closure time, cosmesis, and patient satisfaction as the secondary outcomes.

**Results:**

Of the 278 studies identified, seven randomized controlled trials representing 3705 patients were included in this review. There was no significant difference in SSI rates between sutures and staples in general (OR = 0.98, 95% CI = 0.79–1.22, I^2^ = 44%, P = 0.1) or in a subgroup of gastrointestinal surgery, where subcuticular suturing was found with a comparable SSI risk with skin stapling (OR = 0.85, 95% CI = 0.66–1.09). Staple closure was associated with a shorter surgery duration, whereas sutures appeared to provide better cosmesis and patient satisfaction. Sutures and staples achieved a comparable incidence of dehiscence. There was no significant between-study publication bias.

**Conclusion:**

Our study demonstrated similar outcomes in SSI rate between subcuticular sutures and staples for skin closure in patients undergoing abdominal surgery.

## Introduction

Millions of abdominal procedures are performed annually worldwide [1]. Wound complications are among the most common morbidities for patients after surgery. Surgical site infections (SSIs) are among the major postoperative wound complications that can result in an extended length of stay, additional costs, and a substantial burden to health-care systems [2]. Moreover, patients with SSIs have a 2.2 risk ratio of death compared to those without SSIs, with 75% of death directly attributable to SSIs [3]. Given these consequences, standards and recommendations on postoperative infection prevention have been outlined by the Center for Disease Control and Prevention (CDC) and World Health Organization (WHO) [4, 5]. However, these guidelines do not explicitly address the issue of abdominal wound closure.

Myriad options for skin closure have become available in the last 30 years [6]. A variety of staples and sutures are used in common surgeries such as gastrointestinal, gynaecological, obstetric, urologic, vascular, cardiothoracic, orthopaedic, head, neck, and hand procedures. A German research found that staples were used in 79% of abdominal skin closures, supplemented by absorbable or non-absorbable sutures [7]. Compared with staples, sutures have been demonstrated with superior results on the incidence of wound infections in clean surgical procedures such as the cesarean section [8, 9]. However, the preferred option of skin closure for abdominal surgery remains inconclusive where the wounds are usually exposed to a variety of contributors of surgical site infections, such as endogenous flora from the alimentary or genitourinary tract. A study in the 1980s indicated subcuticular sutures had no advantage over staples in the prevention of incision infection [10], while a retrospective study revealed a significant difference in the incidence of superficial surgical site infection between the stapler group (11.3%) and subcuticular suture group (2.6%) after open hepatobiliary-pancreatic surgery [11]. Therefore, we conducted this meta-analysis to address the limitations of a single study and to establish differences in SSIs between subcuticular staples and sutures in patients undergoing abdominal skin closure.

## Methods

### Search strategy and selection criteria

A comprehensive search in online Medline, EMBASE, and Cochrane Central Register of Controlled Trials via Ovid on January 31, 2020 was conducted for randomized controlled trials (RCTs) comparing staples and sutures after abdominal surgery. A provisional search strategy for the Cochrane Central Register of Controlled Trials can be found in S1 Appendix. We adapted this strategy to search Ovid MEDLINE and EMBASE as well. References were also retrieved to identify additional eligible studies. This systematic search was conducted according to the PRISMA (Preferred Reporting Items for Systematic Reviews and Meta-Analyses) guidelines [12]. The supporting PRISMA checklist of this review is available as supporting information, see S1 Checklist.

Two authors (Feng and Jiang) independently evaluated the articles’ titles and abstracts to decide whether the full text should be further reviewed. In case of disagreement, a consensus was sought through discussion. If this failed, the article would be excluded. We included any randomized controlled studies comparing outcomes of abdominal surgery (elective or emergent, open or laparoscopic) either with subcuticular sutures or staples for skin closure. We excluded ongoing trials and those involving barbed sutures, surgical zippers, skin adhesives, and cesarean delivery.

### Data extraction and principal analysis

Two reviewers (Feng and Jiang) extracted the relevant quantitative and qualitative data necessary for analysis independently. Documented information included authors’ names, year of publication, and patient-related parameters of sex, mean age, sample size, type of surgery, time to staple or suture removal, follow-up, infection, wound dehiscence, cosmesis, closure time, and patient satisfaction.

Our primary outcome was the incidence of SSI after the use of skin staples or sutures in abdominal surgery. No distinctions were made between staples and clips. CDC or WHO standard definitions of SSI were both acceptable for this meta-analysis. The secondary outcomes under investigation included the incidence of wound dehiscence, closure time, cosmesis, and patient satisfaction.

### Statistical analyses

Heterogeneity analysis was performed using the chi-square test and expressed in the I^2^ index. The absence of statistical heterogeneity was indicated by a value of 0%, whereas higher values indicated increased heterogeneity. Pooled odds ratios (ORs) were calculated with 95% confidence intervals (CIs) from a fixed effects model using the Mantel-Haenszel method when there was no evidence of clinical or statistical heterogeneity. Otherwise, a random-effects model was applied. The presence of publication bias was evaluated by using the Cochrane risk of bias tool, which evaluates selection bias (random sequence generation and allocation concealment), performance bias (blinding of participants and personnel), detection bias (blinding of outcome assessment), attrition bias (incomplete outcome data), reporting bias (selective reporting), and other biases between trials. Egger’s test was also used to evaluate publication bias between the studies included. All statistical analyses were conducted using Stata 13.0 (Stata Corporation, College Station, TX) and RevMan Software Version 5.2 (Cochrane Collaboration, Copenhagen, Denmark).

## Results

### Eligible studies

**Fig 1:**
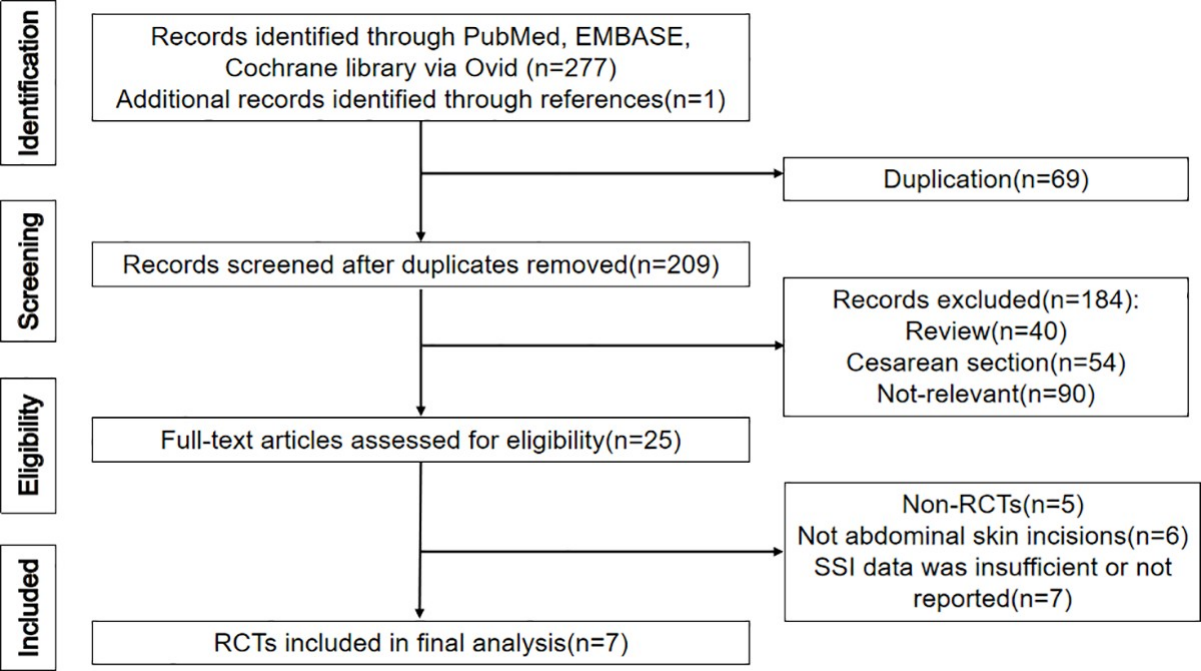
The PRISMA flow chart for the literature search.

The PRISMA flow chart for the literature search is shown in Fig 1. In brief, among the 278 studies identified with the primary search strategy, we excluded 69 duplicated articles and 184 records that were reviews and reports on cesarean skin closure or were irrelevant to the selection criteria. Twenty-five articles were eligible for full-text review. Eighteen of them were further excluded because of the following reasons: non-RCTs (n = 5), non-abdominal surgeries (n = 6), and insufficient or unavailable SSIs data (n = 7). Finally, seven RCTs, published from 1983 to 2019, involving 3705 patients were available for this meta-analysis. A total of 1917 incisions constituted the suture group and 1788 the staple group.

### Study characteristic

The included studies were published from 1983 to 2019, of which three were conducted in Japan [13–15], one in the USA [16], two in the United Kingdom [10, 17], and one in Germany [18]. All the studies provided information on SSIs among patients receiving staples and subcuticular sutures for skin closure. Further evaluation revealed that four were on superficial SSI [13, 15, 16, 18], and three did not specify deep or superficial SSIs [10, 14, 17]. Also, most of the studies used the CDC criteria for SSIs, only one used the WHO criteria [17], and one did not specify the criteria they followed [10]. Four trials included only patients of elective gastrointestinal surgery [13, 14, 17, 18], one had only gynecologic patients [16], and the other two featured heterogeneous populations of elective and emergency, upper and lower gastrointestinal, hepatobiliary and pancreatic, genitourinary and vascular surgery [10, 15]. All of the included studies looked exclusively at open surgery except for one that also characterized laparoscopic surgery [15]. Absorbable sutures were used in five studies, and non-absorbable sutures in two [10, 17]. Only two studies reported the time to staple removal [16, 18], which ranged from 10 to 14 days. The postoperative follow-up duration spanned from 30 days to 6 months. The main characteristics of the enrolled studies are summarized in Table 1.

**Table 1:** Main characteristics of the eligible studies included in the meta-analysis.

Study /Country	Average age (suture/ staple)	Population (suture/staple)	Closure material	Operations	Time to removal (days)	Follow up	Main relevant outcome
Kazuhiro Imamura 2016/Japan	72/73	399 (198/201)	4–0 polydioxan-one sutures, staples	elective or emergency upper or lower gastrointestinal, hepatobiliary and pancreatic (HBP), or vascular surgery	NG	30d	superficial SSI*, postoperative hospitalization;
Toshimasa Tsujinaka 2013/Japan	68/68	1072 (562/518)	3–0 or 4-0polydio-xanone sutures, staples	elective upper or lower gastrointestinal surgery,	NG	30d and 6 months	superficial SSI*, dehiscence, hypertrophic scar formation;
Lindsay M 2017/USA	57/58	163 (79/84)	4–0 absorbable sutures, staples	gynecologic surgery	10–14	30d and 8weeks	wound infection*, dehiscence, scar cosmetic, patient satisfaction, skin closure time;
S.Kobayashi 2015/Japan	65/67	1232 (620/612)	4–0 or 5–0 absorbable sutures, staples	elective colorectal cancer surgery	NG	30d	SSI*, dehiscence, wound aesthetics, patient satisfaction, skin closure time;
Elisabeth Maurer 2019/German	66/61	280 (141/139)	4–0 absorbable sutures, staples	elective gastrointestinal surgery	10	30d	Superficial SSI*, dehiscence, skin closure time;
Joshua Agilinko 2019/United Kingdom	67/69	218 (134/84)	non-absor-bable sutures, staples	elective colorectal surgery	NG	6 weeks and 3 months	wound infection^†^, wound aesthetics, patient satisfaction;
I.R.Pickford 1983/United Kingdom	NG	341 (182/159)	nylon sutures, steel clips	elective and emergency abdominal surgery	NG	NG	wound infection^‡^

Note

* CDC standard

† WHO standard

‡ not specify.

Abbreviation: SSI surgical site infection; NG not given.

### Primary outcome: SSI

**Fig 2:**
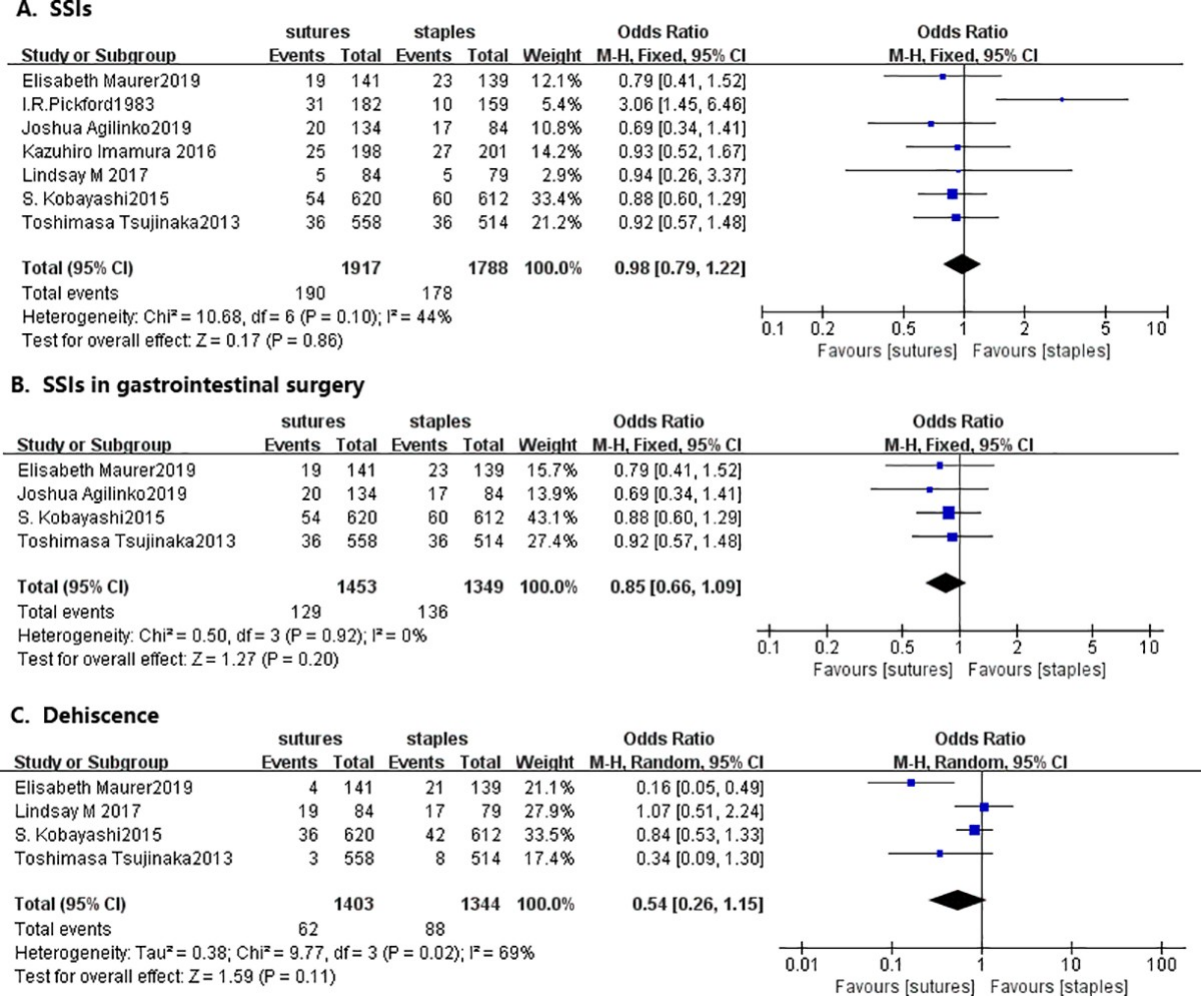
Forest plot of the pooled outcomes of (A) SSIs (B) SSIs in gastrointestinal surgery (C) Wound dehiscence in selected studies comparing staples to subcuticular suture for skin closure after abdominal surgery. Abbreviations: SSIs, surgical site infection; CI, confidence interval; M-H, Mantel-Haenszel.

Data on SSIs were available in all seven studies. As shown in Fig 2A, the difference of SSIs rates between both groups did not reach the significance threshold: 10% (178/1788) in the staple group and 9.9% (190/1917) in the suture group (OR = 0.98, 95% CI = 0.79–1.22, I^2^ = 44%, P = 0.1). The between-group difference was also insignificant when elective gastrointestinal surgery was analyzed alone (OR 0.85, 95% CI = 0.66–1.09, I^2^ = 0%, P = 0.92, Fig 2B).

### Secondary outcomes

#### 1. Wound dehiscence

Data on wound dehiscence were available in four studies, which failed to demonstrate a significant difference between sutures and staples, with a cumulative OR of 0.54 (95%CI, 0.26–1.15, Fig 2C).

#### 2. Closure time

Closure time was the only outcome that favored staples. Table 2 shows the skin closure time reported by three studies, which unanimously presented shorter operating times for staple closure than suture closure. The use of staple was estimated to save 5.5 to 8 minutes.

**Table 2:** Operating time reported in selected studies comparing staples with subcuticular sutures for skin closure after abdominal surgery.

Study	Mean time taken to close skin (min, range)		Estimated time saved by staples (min)
	staples	suture	
Elisabeth Maurer	1.3(0.9–1.8)	7.4(5.3–9.7)	6.1
Lindsay M	3(2–4)	11(9–15)	8
S. Kobayashi	1(0.5–25)	6.5(0.5–30)	5.5

#### 3. Cosmesis and patient satisfaction

Three studies evaluated postoperative cosmesis and patient satisfaction but with different scales of measurement, which rendered a quantitative analysis inapplicable. Instead, qualitative analysis was used, which revealed that sutures were more cosmetically desirable in two trials but comparable with staples in the third study. Thus, we concluded that sutures have a cosmetic advantage over staples. Similarly, subcuticular sutures have the edge over staples in patient satisfaction (Table 3).

**Table 3:** Qualitative data of outcomes of selected studies comparing staples with subcuticular sutures in the postoperative cosmesis and patient satisfaction.

Study	Patient satisfaction	Cosmesis
Lindsay M	equivalent	suture superior
S. Kobayashi	suture superior	equivalent
Joshua Agilinko	suture superior	suture superior
Conclusion	suture superior	suture superior

### Risk of bias assessment for included studies

**Fig 3:**
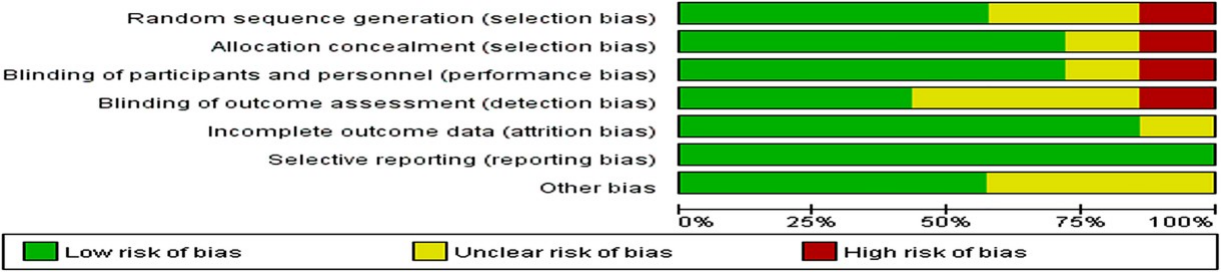
Risk of bias.

Fig 3 shows an overview of the risk of bias assessment according to the Cochrane risk of bias tool. Blinding was absent in one of the seven trials, which could have biased the detection of SSIs. In the trial by Joshua Agilinko et al, a high risk of bias was introduced by inadequate randomization. Egger’s test (P = 0.11) indicated no publication bias in any of the seven studies.

## Discussion

The most recent guidance from the National Institute for Health and Clinical Excellence (NICE) for the prevention and management of surgical site infection [19] has addressed the issue of suturing techniques and the risk of surgical site infections for the first time. It is also the first time that it recommends using sutures rather than staples to close the skin after the cesarean section to reduce wound dehiscence among post-partum women. However, evidence that favors sutures over staples has been limited when it comes to other types of surgery. This meta-analysis does not support the risk-reduction effect of either subcuticular sutures or staples in infection and wound dehiscence after abdominal incision closure.

The impact of different closure techniques on surgical site infections varies depending on the anatomical sites. A similar infection rate in suture and staple group (2 and 3%) has been reported in craniofacial surgery [20]. In orthopaedic surgeries, two previous meta-analyses comparing staples versus sutures have led to conflicting conclusions on the relative risks for surgical site infection between skin closure methods [21, 22]. However, based on an updated meta-analysis, no definite differences in SSI risk were found between staples and sutures [23]. Data from a Cochrane review has also provided similar results in coronary artery bypass surgery [24].

The inconsistent evidence could be interpreted as reasonable since both sutures and staples have their strength and weakness. Metal staples are believed to be less irritative to human tissue and more resistant to infection than the least reactogenic suture [25]. Moreover, as a faster option of skin closure with approximately 5.5 to 8 minutes saved during a procedure, staples are preferred in the emergency setting. Disadvantages of staples include the potential for staple track formation, damage of hair follicles, sweat and sebaceous glands, bacterial migration into the wound bed, and discomfort during staple removal [26]. Considering that the patient’s flora is the primary source of infection at the surgical site, a tight skin closure that preserves the dermal structure may help prevent surgical site infection. Intracutaneous sutures are intended to close the epidermis tight without damaging dermal structures. Also, patients may find absorbable sutures more comfortable as it can be left in the wound without removal. What’s more, sutures cost only one-fifth of staples [27]. However, all suture materials are foreign bodies to human tissue and may cause tissue reactions, such as an inflammatory response, which may lead to compromised wound healing and elevated risk of infection [6]. Intriguingly, risks and benefits aside, surgeons lean towards staples for closing midline incisions [7].

One of the lasting and conspicuous reminders of any abdominal surgery is the scar at the site of the incision [26]. Since cosmesis is of particular interest in gynaecological practice than in general surgery [28], we evaluated the cosmesis and patient satisfaction between staples and sutures. Given that quantitative measurement was not an option due to inconsistent scales adopted in the included studies, unfortunately, we concluded from the qualitative analysis that sutures were preferable to staples regarding cosmetic effects and patient satisfaction. However, our findings should be interpreted with caution. A study of mature post-laparotomy scars at least one year out revealed that the patients’ overall impression of the wound favored a sutured closure with a smaller scar area (and free from staple marks) than staples. Hence, patients who care about skin marks would benefit more from subcuticular skin closure. Besides, no other differences were noted in the self-assessment of pain, itching, color, hardness, thickness, and irregularity of the scars [29]. Similarly, the study by Obermair et al. [30] analyzed patients undergoing open gynaecological surgery and concluded that the cosmetic effects of staples were no better than sutures after surgery. Interestingly, in the subgroup analysis of types of incisions, staples produced a less desirable cosmetic result than subcuticular in transverse abdominal wounds but not in vertical wounds [31], leaving the decision of skin closure option a matter of surgeon preference.

However, the results of this meta-analysis should be interpreted along with their limitations. Six out of the seven studies included looked exclusively at open surgeries. Therefore, we have scarce data on the impact of sutures or staples on laparoscopic procedures. In fact, each trial involved multiple types of abdominal procedures, but we were unable to perform subgroup analyses by the types of surgery due to the lack of relevant information. Consequently, the relationship between skin closure techniques and patient outcomes in different kinds of surgeries still warrants investigation.

Based on a limited number of eligible studies, this analysis is not representative of all populations. For example, obesity is believed to elevate the risk of SSI in many ways: malnutrition, demanding exposure during surgery and the resultant longer operating time, inadequate oxygenation of tissues, and decreased antibiotic penetration [32]. Important it is, though, BMI was only available in two studies. As a result, the contribution of obesity status was not evaluable. Similarly, other factors that may affect wound complications include advanced age, diabetes mellitus, malnutrition, smoking, immunosuppressive medications, and several others summarized by Fry [33]. However, their effects on wound complications were not accounted for in any of the included studies.

Moderately significant heterogeneity was noted between the RCTs. They varied in the types of surgery, time to device removal and length of follow-up, suture materials, elective versus emergency cases, and different criteria and definition of wound infection. Fortunately, the statistical heterogeneity could be explained by using subgroup analyses as the I^2^ for SSI heterogeneity was 0% when data were sub-divided by gastrointestinal surgery. This result indicated that surgery types were the primary source of heterogeneity.

In conclusion, our study showed that skin closure with subcuticular sutures seems comparable to staples in preventing SSI in abdominal surgeries. As surgeons need better evidence for decision-making, well-designed randomized controlled trials are warranted to validate the results of this meta-analysis. Also, the role of risk factors known to be associated with postoperative complications, such as obesity, should be addressed in future studies.

## Supporting information

S1 Checklist PRISMA checklist. (DOC)

S1 Appendix Search strategy. (DOCX)
