# Supplementary material for: Subcuticular sutures versus staples for skin closure in patients undergoing abdominal surgery: A meta-analysis of randomized controlled trials
Source: PLoS One. 2021 May 4;16(5):e0251022. doi: 10.1371/journal.pone.0251022 (PMC8096075; doi:10.1371/journal.pone.0251022)
Supplement: S1 Appendix — (DOCX) [file pone.0251022.s002.docx]

#1 MeSH descriptor: [Sutures] explode all trees

#2 MeSH descriptor: [Surgical Staplers] explode all trees

#3 MeSH descriptor: [Surgical Stapling] explode all trees

#4 #2 or #3

#5(subcutaneous or sub-cutaneous or subcuticular or sub-cuticular):ti,ab,kw

#6 #1 and #5

#7#4 and #6

#8((sutur* or “hand sewn” or “hand sewing” or stitch* or handsewn

or “manual closure” or catgut* or “cat gut”) and stapl*):ti,ab

#9 #7 or #8

#10 MeSH descriptor Surgical Wound Infection explode all trees

#11MeSHdescriptor Surgical Wound Dehiscence explode all trees

#12 (surg* near/5 infect*):ti,ab,kw

#13 (surg* near/5 wound*):ti,ab,kw

#14 (surg* near/5 site*):ti,ab,kw

#15 (surg* near/5 incision*):ti,ab,kw

#16 (surg* near/5 dehisc*):ti,ab,kw

#17 (wound* near/5 dehisc*):ti,ab,kw

#18 (wound* near/5 infect*):ti,ab,kw

#19 (wound near/5 disruption*):ti,ab,kw

#20 (wound next complication*):ti,ab,kw

#21 #10 or #11 or #112 or #13 or #14 or #15 or #16 or #17 or #18

or #19 or #20

#22 #9 and #21
